# Technical challenges and limitations of current mouse models of ovarian cancer

**DOI:** 10.1186/1757-2215-5-39

**Published:** 2012-11-29

**Authors:** Kenneth Garson, Lisa F Gamwell, Elizabeth MG Pitre, Barbara C Vanderhyden

**Affiliations:** 1 Centre for Cancer Therapeutics, Ottawa Hospital Research Institute, Ottawa, ON K1H 8L6, Canada; 2 Department of Cellular and Molecular Medicine, Faculty of Medicine, University of Ottawa, Ottawa, ON K1H 8M5, Canada

**Keywords:** Ovarian cancer, Mouse models, Genetically engineered, Ovary, Oviduct, Ovarian surface epithelium

## Abstract

The development of genetically engineered models (GEM) of epithelial ovarian cancer (EOC) has been very successful, with well validated models representing high grade and low grade serous adenocarcinomas and endometrioid carcinoma (EC). Most of these models were developed using technologies intended to target the ovarian surface epithelium (OSE), the cell type long believed to be the origin of EOC. More recent evidence has highlighted what is likely a more prevalent role of the secretory cell of the fallopian tube in the ontogeny of EOC, however none of the GEM of EOC have demonstrated successful targeting of this important cell type.

The precise technologies exploited to develop the existing GEM of EOC are varied and carry with them advantages and disadvantages. The use of tissue specific promoters to model disease has been very successful, but the lack of any truly specific OSE or oviductal secretory cell promoters makes the outcomes of these models quite unpredictable. Effecting genetic change by the administration of adenoviral vectors expressing *Cre* recombinase may alleviate the perceived need for tissue specific promoters, however the efficiencies of infection of different cell types is subject to numerous biological parameters that may lead to preferential targeting of certain cell populations.

One important future avenue of GEM of EOC is the evaluation of the role of genetic modifiers. We have found that genetic background can lead to contrasting phenotypes in one model of ovarian cancer, and data from other laboratories have also hinted that the exact genetic background of the model may influence the resulting phenotype. The different genetic backgrounds may modify the biology of the tumors in a manner that will be relevant to human disease, but they may also be modifying parameters which impact the response of the host to the technologies employed to develop the model.

## Review

### Introduction

Recently a number of insightful reviews have summarized GEM of EOC [1-9]. This review will first briefly examine current hypotheses on the cell(s) of origin of human EOC, as this is the cornerstone for the development of GEM of EOC. Secondly, the numerous models of ovarian cancer will be briefly discussed in the context of the technological approaches exploited for model development. Importantly, the advantages, disadvantages and inherent assumptions implicit in these technologies will be discussed. Finally, we have found that the genetic background can impart dramatic changes in the phenotype of disease in a GEM of EOC and the latter part of this review will discuss the relevance of genetic background and inbred strains to future model development.

The fundamental premise in the design of GEM of EOC is the development of tools or strategies to target genetic change to the presumed precursors of the disease. Traditionally, the origin of human EOC was presumed to be in the OSE, or in the epithelial lining of inclusion cysts that had arisen from the OSE and the evidence supporting this has been extensively reviewed [10-14]. The OSE retains the potential to undergo Müllerian differentiation as evidenced by the expression of Müllerian markers in naturally forming inclusion cysts, in inclusion cysts arising at increased frequencies in mice harboring specific genetic lesions [15,16] and in OSE coaxed into different Müllerian lineages following the introduction of specific *Hoxa* genes [17].

Recently, growing evidence has challenged this theory and identified the secretory cell of the distal fallopian tube as a putative EOC precursor [18-25]. While evidence favors a fallopian tube origin for a high proportion of high grade serous ovarian adenocarcinomas, there has not been sufficient evidence to rule out a parallel role for the OSE [25,26].

Finally, it must be kept in mind that evidence of early precursors of EOC in the OSE or the secretory cell of the distal fallopian tube implicates not only these differentiated cell types as potential origins of the disease, but it also implicates putative progenitor or tissue stem cells as well [27-32].

### Targeting the mouse for models of ovarian cancer

#### Direct expression of a transgene from a tissue specific promoter

In the absence of a promoter candidate specific for OSE, Connolly et al. [33] found that the *Amhr2* (also known as MISIIR) promoter was transcriptionally active in murine OSE in addition to reported expression in granulosa cells [34] and the stroma of the Müllerian duct [35]. In the first GEM of EOC, female mice expressing the SV40 early region (SV40TAg) from the *Amhr2* promoter developed poorly differentiated serous tumors derived from the OSE. Interestingly, granulosa cell tumors did not arise in this model, despite the potential for the *Amhr2* promoter to drive expression in granulosa cells [34,35] and the potential for granulosa cells to become malignantly transformed by the SV40 early region [36,37] or the SV40 large T antigen (TAg) [38].

**Figure 1:**
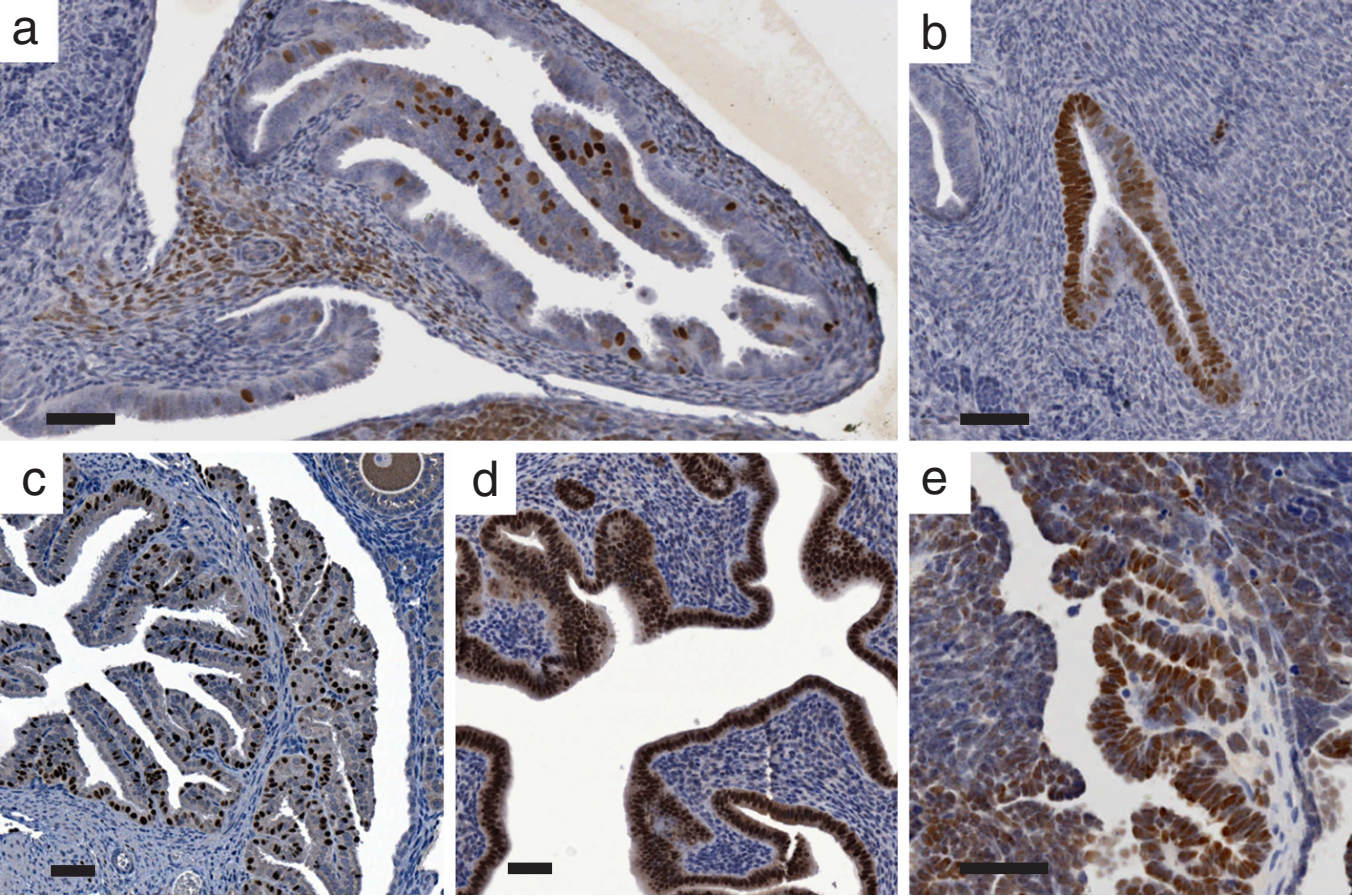
**Expression of TAg and PAX8.** TAg in the oviductal epithelium and stroma (**a**) and in the uterine epithelium (**b**) of FVB/n-Tg(Amhr2-SV40TAg)1Bcv mice as shown by immunohistochemistry. PAX8 expression in the normal oviduct (**c**), uterine epithelium (**d**) and papillary lesions of a tumor from an ovariectomized FVB/n-Tg(Amhr2-SV40TAg)1Bcv mouse (**e**). Scale bars represent 50 μM.

In our laboratory, we noted in an independent Amhr2-SV40TAg transgenic line (Tg(Amhr2-SV40TAg)1Bcv) generated on the FVB/n strain^a^, that in addition to evident TAg expression in the ovary, expression was also seen in the epithelium and stroma of the oviduct (Figure 1a) and in the epithelium of the uterus (Figure 1b). Expression of TAg in the uterine and oviductal epithelium was not expected from the endogenous *Amhr2* promoter and may result from altered regulation of the ectopic promoter fragment. In order to examine potential tumorigenesis from the oviduct and uterus, ovaries were removed from mice 7–8 weeks of age. Tumors arising from the remaining reproductive tract were slower to develop and showed significantly more cells expressing PAX8 than tumors developing from the ovaries of the non-ovariectomized controls. Expression of PAX8, a marker of oviductal and uterine epithelium (Figure 1c, 1d, respectively), in tumors from ovariectomized mice may indicate their origin in the oviductal or uterine epithelium. Interestingly, several regions of papillary differentiation were found in tumors with PAX8 positivity (Figure 1e).

Direct targeting of SV40TAg expression to the oviductal secretory cells was attempted using the oviduct-specific glycoprotein (*Ovgp1*) promoter [39]. Although oviductal tumors were reported they were not well characterized [39]. All mice developed uterine tumors and 60% developed vaginal tumors, two other tissues with documented OVGP1 expression [40,41].

Other relevant models have induced premalignant changes, but not cancers, in the OSE and have implicated various signaling pathways in these changes. Expression of a constitutively active PIK3CA by the *Amhr2* promoter led to OSE hyperplasia [42]; the expression of dnSMAD2 from the *Amh* promoter gave rise to ovarian inclusion cysts with Müllerian differentiation [43] and OSE in mice heterozygous for disabled-2 demonstrated surface dysplasia and papillomatosis [44].

While the direct transgene approach led to the first model of EOC, the implementation of such strategies remains unpredictable. Both the *Amh* and *Amhr2* promoters express in both granulosa cells and OSE [33,43], yet their use to express the SV40TAg has led to granulosa cell tumors [38] and epithelial tumors [33], respectively. It is possible that there are subtle differences not only in the static expression from a promoter at a fixed time point, but also differences in the developmental timing of expression that might be critical for the phenotype of the model. It must not be forgotten that the expression of oncogenes in the earliest cell types that initiate promoter function may also alter the development of subsequent lineages of cells. This is in contrast to our notions of tumorigenesis, where dominant genetic changes occur stochastically in cells residing in organisms that for the most part have completed their developmental programs.

#### Cre-lox conditional targeting: Tissue expression of Cre

The first model exploiting the tissue-specific expression of *Cre* recombinase used the *Fshr* promoter to inactivate loxP-flanked (floxed) *Brca1* alleles in granulosa cells, however serous cystadenomas carrying unrecombined *Brca1* developed in the OSE [45]. Increased expression of aromatase [46], and alterations of the estrus cycle [47] due to the inactivation of *Brca1* may have collectively led to increased levels of estrogen [47] contributing to the observed changes in the OSE. While this model may seem unique, other examples in the female reproductive tract of genetic change in one compartment leading to hyperplasia or tumorigenesis in a second compartment have been reported recently [48,49].

The *Amhr2* promoter, used for the first GEM of EOC, has been exploited for the expression of *Cre* in a number of models. Expression of *Cre* from the endogenous *Amhr2* promoter leads to conditional expression of *LacZ* in the *Gtrosa26*^*tm1Sor*^ reporter mice as early as embryonic day 11.5 in female urogenital ridges, and in the female gonads and Müllerian ducts at embryonic day 12.5. In the adult mouse, expression of *LacZ* has been noted in the granulosa cells of follicles (not primary or primordial), thecal cells, corpus luteum, OSE and stromal cells of the oviduct and uterus [50]. Importantly, *LacZ* expression has not been reported in the oviductal or uterine epithelium.

The first low grade EOC model was reported by Fan et al. in 2009 [51]. Briefly, while examining the role of KRAS^G12D^ and PTEN signaling in granulosa cell development, they noted in triple transgenic mice (*Ptenfl/fl;KrasG12D;Amhr2-Cre*) that the loss of *Pten* and gain of *Kras*^*G12D*^ in the OSE led to the development of low grade serous adenocarcinomas. Activation of *Kras*^*G12D*^ alone by *Amhr2-Cre* did not lead to tumorigenesis, but rather growth arrest of granulosa cells and OSE hyperplasia [52]. Conditional loss of *Pten* alone in *Amhr2-Cre* mice resulted in alterations in granulosa [53,54] and uterine myometrial cells [55] but not OSE.

The OSE have also been targeted for alteration of the WNT pathway through the activation of β-catenin following the conditional deletion of exon 3 (*Ctnnb1*^*Δex3*^) in *Amhr2-Cre* mice [56]. While all mice developed pre-tumoral nests of cells at the ovarian surface, 50% of mice progressed to develop EC. Combining the conditional activation of *Ctnnb1*^*Δex3*^ with the conditional inactivation of *Pten* led to all mice developing EC. The phenotype of this model is in contrast with other reports that described the development of granulosa cell tumors in mice with conditional loss of *Pten* and conditional activation of β-catenin (*Ctnnb1*^*Δex3*^) induced either in the *Amhr2-Cre* line or through granulosa specific expression of *Cre* in the *Cyp19Cre* (Tg(CYP19A1-cre)1jri) line [57]. It is unclear from the reports [53,56,57] whether the discrepancies relate to different interpretations of the pathological findings or whether possible differences in the breeding strategies to achieve the triple transgenic model animals led to different genetic backgrounds and outcomes.

The third transgenic model of EOC generated using the *Amhr2-Cre* transgenic strain was achieved through the conditional inactivation of *Dicer* and *Pten*, two genes frequently down-regulated in ovarian cancer [58]. While the phenotype of *Amhr2-Cre* mediated deletion of *Pten* is described above, the conditional deletion of *Dicer* alone resulted in smaller oviducts and uterine horns, a disorganized oviduct, changes in the glandular structure of the uterus [59] and alteration of ovarian follicle pools [60]. The combined conditional inactivation of both *Dicer* and *Pten* led to the development of high grade serous adenocarcinomas from the oviduct.

In contrast to evidence suggesting an origin for human ovarian cancer in the epithelium of the fallopian tube, Kim et al. identified early lesions in the stroma of the oviduct, consistent with the expression pattern from the *Amhr2* promoter in this tissue [58]. These lesions and the resulting tumors expressed epithelial markers with gene expression patterns which aligned with human high grade serous ovarian carcinomas. The relationship of these early stromal lesions to the presumed fallopian tube secretory cell precursors of human disease is not clear. In the mouse, oviductal epithelial cells are not derived from stromal cells which express AMHR2 [61]. It would be of interest to determine if rare epithelial cells reside in the stroma of wild type mice or whether the loss of tumor suppressors in the oviductal mesenchyme leads to the ectopic development of epithelial precursors.

In summary, the targeting of genetic change through the inactivation of tumor suppressors (*Brca1, Pten, Dicer*) or the activation of oncogenes (*Kras*^*G12D*^, *CtnnB1*) using the tissue specific expression of *Cre* recombinase has led to EOC models encompassing benign tumors, EC, low grade serous and high grade serous disease, however, there remain a number of caveats. First, the transient expression of *Cre* from a tissue specific promoter will mark that cell and all subsequent lineages with the designed genetic change, irrespective of whether the promoter driving *Cre* has ceased to be transcriptionally active. This may exacerbate concerns about the loss of tumor suppressors or gain of oncogenes leading to models of “developmental cancer”.

A second caveat of knock-in strains expressing *Cre* recombinase is dependent on the design. Transgenic lines such as the *Amhr2-Cre* line, which create a null allele [50], are used experimentally as heterozygotes. While no reports indicate any possible phenotypic consequences of haploinsufficiency of the *Amhr2* gene, it remains a theoretical possibility that this may shape the phenotype of the resulting models. A phenotype based on haploinsufficiency was reported for *Foxg1-cre* mice [62] in which altered cell populations and tissue structure were observed in the brain of heterozygous mice. Further seemingly innocuous facets of the experiment can also lead to surprising phenotypic consequences. Mice carrying unrecombined floxed alleles can manifest phenotypes distinct from the wild type [63,64], and strains of mice expressing *Cre* from tissue specific promoters can present altered phenotypes in the absence of any floxed target genes [65].

#### Cre-lox conditional targeting: Viral expression of Cre

Adenoviral vectors expressing *Cre* recombinase (*Ad-CMV-Cre)* have been used for GEM of EOC to control both the time and location of *Cre* expression. Injection of virus through the bursal membrane or infundibulum has been used primarily to alter gene expression in the OSE, however this route also permits exposure of the bursal membrane and the oviductal epithelium to administered virus. The accessibility of the oviductal epithelium to introduced virus can clearly be seen in Figure 1c, which shows the externalized epithelium of the oviductal infundibulum, with secretory cells (PAX8 expressing) within 30 μM of the OSE.

The first model of EOC using the intrabursal administration of *Ad-CMV-Cre* reported high grade serous ovarian adenocarcinomas in mice that had conditional loss of *Trp53* and *Rb1* alleles following *Cre*-mediated recombination [66]. Interestingly, subtle variations in methodology may impact the phenotypes of this model, as different results have been noted in different laboratories. Clark-Knowles et al. found the loss of *Trp53* and *Rb1* alleles using intrabursal administration of *Ad-CMV-Cre* led to the development of leiomyosarcomas, a phenotype that was also seen with 100% penetrance in mice with conditional deletion of *Trp53* alleles alone [67]. In contrast, Flesken-Nikitin only observed the rare appearance of serous adenocarcinomas in *Trp53*^*loxP/loxP*^ mice treated with *Ad-CMV-Cre*[66]. A third report by Quinn et al. observed the rare occurrence of leiomyosarcomas in mice with conditional loss of *Trp53*. Possible differences in methodology, including different mouse genetic backgrounds, may have caused the variable outcome of these experiments. Novel in the experiments of Quinn et al. and Clark-Knowles et al. was a more aggressive or more penetrant disease when *Brca1* was deleted in addition to loss of *Trp53*[67,68], a finding also noted for uterine leiomyosarcomas [69].

Clark-Knowles et al. (2009) speculated that one possible source of the leiomyosarcomas was from the smooth muscle cells of the bursal membrane. Recently, Szabova et al. [70] re-examined the role of *Rb1, Trp53, Brca1* and *Brca2* in EOC applying several new innovations that addressed two concerns. First, the concern that deletion of *Rb1* alone may be ineffective due to functional compensation by *p107* or *p130*[71] led the authors to abrogate *Rb1, p107* and *p130* function through the *Cre*-dependent conditional expression of a deletion mutant of the SV40 Large T antigen, T_121_[72]. T_121_ binds and inactivates RB1 and related proteins p107 and p130 [72,73] but does not inactivate TRP53. Furthermore, to eliminate the potential transformation of smooth muscle cells in the bursal membrane, conditional expression of T_121_ was from the cytokeratin 18 promoter. In contrast to the previous reports [66,67], inactivation of RB1 (and p107, p130) following activation of the T_121_ led to a range of abnormalities in the OSE including serous EOC (often high grade) in 18% of mice. Single or double loss of *Trp53, Brca1* or *Brca2* function failed to generate OSE pathology while loss of *p53* alleles in conjunction with expression of T_121_ led to both the increased frequency and progression of high grade serous EOC [70].

In a related model, intrabursal administration of *Ad-CMV-Cre* was used to activate expression of the SV40 early region in mouse OSE [74]. The tumors emerging from this model were described as sex cord stromal tumors. While the SV40TAg is able to inactivate both RB1 (and p107, p130) and TRP53, additional roles in transformation have been described for both the large and small t antigens [75] and how each of these contribute to the described phenotype is not known. Interestingly, treatment of mice with 17β-estradiol led to an earlier onset of tumors, decreased survival and distinctive papillary histology [74].

While targeting of OSE using the *Amhr2-Cre* transgenic to activate *Kras*^*G12D*^ and to inactivate *Pten* resulted in low grade serous adenocarcinomas [51], similar activation/inactivation of these genes induced following the intrabursal injection of *Ad-CMV-Cre* led to the development of ovarian endometrioid adenocarcinomas [76]. While both of these reports indicate tumors arising from the OSE, the two different strategies may target different subsets of cells on the ovarian surface. Alternatively, the different outcomes in these two experiments may reflect the activation/deletion of these genes at different times in development. Inactivation of *Pten* alone following the intrabursal administration of *Ad-CMV-Cre* has been reported to lead to the development of endometrioid lesions [76] or serous papillary hyperplasia [77], again a variable outcome depending on the reporting laboratory.

Activation of *Kras*^*G12D*^ has also been evaluated in conjunction with the conditional loss of the *Trp53* allele following intrabursal delivery of *Ad-CMV-Cre*[78]. While the resulting tumors were described as sarcomatoid ovarian carcinomas, the authors illustrated the utility of a more stochastic model of tumor initiation for understanding the evolution of immune cell infiltrates in the tumor microenvironment. Importantly, the authors reported that tumor growth is controlled initially by immune surveillance and the emergence of clinically significant tumors results from the development of an immunosuppressive tumor microenvironment.

Other investigators have addressed the loss of *Pten* in conjunction with further insult to the *Akt* pathway [79]. Interestingly, while one of the primary interests in the inactivation of *Pten* is to activate the *Akt* pathway, Kinross et al. inactivated *Pten* and activated a constitutively active mutant *Pik3ca* allele (Pik3ca*) expressed from the *Pik3ca* endogenous promoter. While the single lesions alone led to serous papillary hyperplasia, the combined loss of *Pten* and expression of Pik3ca* led to the development of high grade serous ovarian cancer.

Loss of PTEN function has also been coordinated with activation of the *Wnt* signaling pathway [80,81]. Wu et al. reported a statistical association of *Pten* mutations with *Wnt* pathway defects in ovarian endometrioid adenocarcinomas in clinical samples and used *Ad-CMV-Cre* to activate the *Wnt* pathway by deletion of *Apc* and the *Akt* pathway by deletion of *Pten* to give rise to ovarian endometrioid adenocarcinomas in 100% of mice. Interestingly, activation of the *Akt* and *Wnt* signaling pathways was also reported using *Amhr2-Cre* to inactivate *Pten* and activate the expression of a dominant stable β-catenin [53,56], however depending on the reporting laboratory, the mice either developed EC [56] in agreement with Wu et al. or granulosa cell tumors [53].

In summary, the administration of *Ad-CMV-Cre* intrabursally has led to models of both high grade serous and endometrioid adenocarcinomas. While *Ad-CMV-Cre* models require surgical intervention to initiate the tumorigenic process, the sudden induction of genetic mutation in the target cells in the adult is more relevant to the human disease than a developmentally regulated acquisition of genetic change. The exposure of the ovarian surface, bursal membrane, oviduct and in some instances granulosa cells [77] to intrabursally injected adenovirus represents a simple model of delivery of *Cre*, however the efficient infection of polarized epithelium of the oviduct and even polarized OSE [82] is much more complicated.

The successful targeting of a cell for expression of *Cre* recombinase from *Ad-CMV-Cre* vectors depends on the activity of the CMV promoter in that cell type and the level of expression of the virus receptor and co-receptors, CAR [83] and the αvβ3 or αvβ5 integrins [84], respectively. In polarized epithelium, infection can be restricted to the basolateral surface of the cell due to the absence of apically expressed CAR [85,86]. A number of factors can sensitize cells to apical infection, including the apical expression of alternate isoforms of CAR [87], and the expression of extrinsic factors such as CXCL8 [88] or lactoferrin [89]. Expression of both CXCL8 [90,91] and lactoferrin [92,93] has been reported in follicular fluid and within the fallopian tube. While successful infection of OSE is evident in the reported models, it cannot be assumed that all cells are equally sensitive to infection.

**Figure 2:**
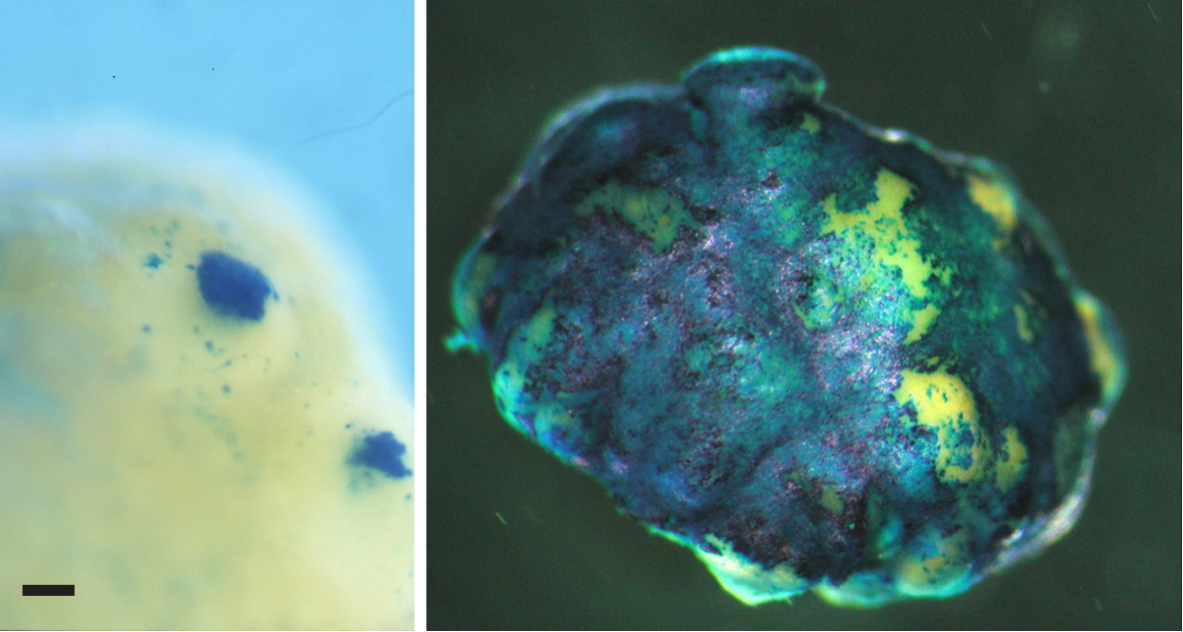
***LacZ *expression in *Ad-CMV-lacZ *treated ovaries.** Intrabursal administration of 10^7^ (left panel) or 10^8^ (right panel) plaque forming units (pfu) of *Ad-CMV-lacZ*. Administration of 10^7^ pfu results in a selective infection of a subset of cells on the surface of the murine ovary (left). Ovaries were fixed and stained with X-gal to detect expression of β-galactosidase. The scale bar on the left panel indicates approximately 150 μM.

Previously, we have examined infection of the OSE following the intrabursal administration of varying titres of *Ad-CMV-/acZ*. Although infection, measured by LacZ expression, was at times found throughout the entire ovarian surface, a subset of mice showed infection localized frequently at the apex of what appear to be corpora luteal structures (Figure 2). The reasons for such localized *LacZ* expression on the epithelial surface are not clear, but it could result from a number of mechanisms, including the selective expression of *LacZ* from the CMV promoter resident in the adenoviral vector or the selective infection of cells on the ovarian surface that had lost polarity during ovulatory wound repair.

While empirical data from our laboratory and others have indicated that adenovirus can infect the epithelium of the ovary, oviduct and uterus [94], it remains a concern that subsets of cells may have varied sensitivities to infection which may influence the phenotype of the resulting models.

### Genetic background

**Figure 3:**
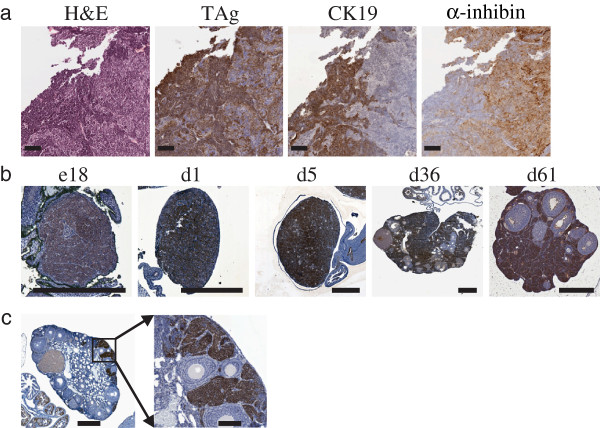
**Effect of genetic strain on ovarian tumors in Tg(Amhr2-SV40TAg)1Bcv mice.** Cytokeratin 19 (CK19) and α-inhibin expression in the FVB/n-Tg(Amhr2-SV40TAg)1Bcv (**a**) are localized to distinct regions of the tumor that appear mutually exclusive. TAg expression is found throughout the entire ovarian stroma as early as embryonic day 18 (**b**) with exclusion of expression from large follicles apparent at 36 and 61 days of age. Expression of TAg in the F1 ovaries from a cross of the FVB/n-Tg(Amhr2-SV40TAg)1Bcv to C57Bl/6 mice is apparent after several weeks of age and then only localized to the ovarian cortex as shown (**c**, day 43). Scale bars indicate 100 μM (a), 500 μM (b + c) or 100 μM (c, enlarged area).

The importance of mouse strain on the investigation of transgenic models of cancer has been recently reviewed [95]. Our laboratory found striking phenotypic differences in a transgenic line, Tg(Amhr2-SV40TAg)1Bcv, depending on whether it was in a pure FVB/n or a mixed FVB/n;C57Bl/6 background (FVBB6F1). Ovarian tumors arising in Tg(Amhr2-SV40TAg)1Bcv and independent FVB/n transgenic founders showed regional expression of both cytokeratin and α-inhibin that tended to be mutually exclusive (Figure 3a). In the FVB/n line, stromal hyperplasia of TAg expressing cells is evident as early as embryonic day 18 (Figure 3b). In contrast , F1 progeny of a backcross into the C57/Bl6 strain revealed TAg expressing lesions only in the cortex of the ovary near the ovarian surface as late as 6 weeks of age (Figure 3c), a phenotype which is distinct from that observed in the pure FVB/n background. Finally, the delayed initiation of ovarian cancer in the FVBB6F1 mice was reflected in a longer mean time to endpoint of 21 weeks compared to 15 weeks observed for the Tg(Amhr2-SV40TAg)1Bcv in the pure FVB/n background.

A number of the EOC models described in this review have targeted similar pathways with different consequences. The modeling of *Trp53* and *Rb1* loss following the intrabursal delivery of *Ad-CMV-Cre* was performed by two laboratories with different outcomes [66,67]. The penetrance and phenotype of tumors arising in *Ad-CMV-Cre* treated animals with only loss of *Trp53* was also varied between reports [66-68]. While these groups initially used the same strains of mice, Flesken-Nikitin et al. reported the backcross of their strains to the FVB/n background. Although not verified, the varied phenotypes in these studies may be a consequence of different genetic backgrounds.

One future goal for the continued development of GEM of EOC is to identify modifiers of the disease through crosses with genetically varied strains of mice. While libraries of recombinant inbred strains have been developed for this purpose [96], the requirement of many of the EOC models for three transgenes and a requirement for homozygosity of floxed tumor suppressor alleles makes the breeding for such a systematic approach challenging. In addition, the use of GEM of EOC to screen for genetic modifiers of disease that are relevant to human will require sorting through “noise” which is related to genetic modifiers of “technology” as opposed to modifiers of the disease. For example, strain differences may modulate parameters that influence the efficiency of viral infection, or subtly modify temporal patterns of promoter function.

## Conclusions

**Figure 4:**
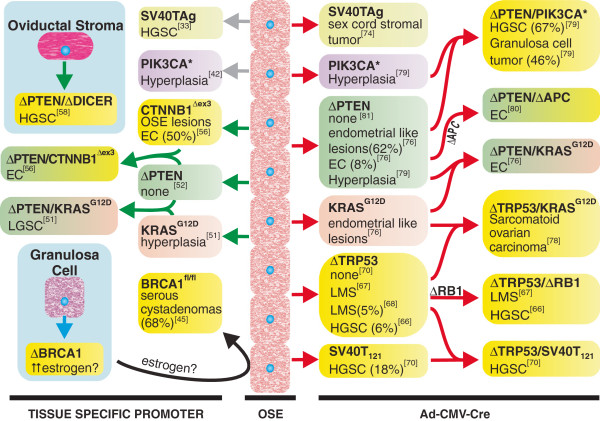
**GEM of EOC.** Outline of current models of EOC originating from genetic modification of OSE (centre), granulosa cells (bottom left) and oviductal stroma (top left). Phenotypes of OSE carrying single genetic lesions induced by *Ad-CMV-Cre* (red arrows) or tissue specific expression of oncogenes using the *Amhr2* promoter (gray arrows), or expression of *Cre* from the FSHR promoter (blue arrow) or expression of *Cre* from the endogenous *Amhr2* promoter (green arrows) are indicated. Murine models elicited phenotypes consistent with high grade serous ovarian cancer (HGSC), low grade serous ovarian cancer (LGSC), endometrioid ovarian cancer (EC), leiomyosarcomas (LMS), or the indicated phenotypes. Penetrance of the noted phenotypes, when less than 80%, is expressed as the percentage of animals affected and displayed in brackets. Phenotypes of models are presented in yellow backgrounds, however models based on the activation/inactivation of similar pathways by separate means (eg. *Ad-CMV-Cre* versus *Amhr2-Cre*) are shaded with the same colors to facilitate comparison.

This review has examined some of the technological parameters that have shaped the existing GEM of EOC. The modeling of EOC has been very successful, with well validated models representing high grade serous adenocarcinoma [33,58,66,70,79], low grade serous adenocarcinoma [51], and endometrioid carcinoma [56,76,80] (Figure 4). Eight GEM of EOC derived from OSE adds weight to the notion that the OSE retains a plasticity that does not preclude malignant transformation into cancers with evident Müllerian differentiation. The lack of GEM of EOC representing cancers arising primarily from the oviductal epithelium may be a consequence of inefficient targeting strategies.

Overall, the development of GEM of human cancers is a balance between convenience and authenticity. By some measures, the ultimate GEM of EOC would be a transgenic model requiring no intervention yet yielding 100% penetrance in mature animals of a phenocopy of the human disease with a predictable onset and highly consistent endpoint. While useful for statistical analysis of survival in therapeutic trials, this type of model is in stark contrast to the human disease which arises stochastically, in varied genetic backgrounds with highly variable rates of progression.

Now that some excellent models of EOC have been derived, it is time to develop models that may be less predictable, yet designed with imaging in mind such that 100% penetrance and highly defined endpoints are not essential. Consistent with the development of more stochastic models of ovarian cancer, GEM which exhibit genomic instability [68,97], a prominent feature of high grade serous EOC, should be important for future model development. Importantly, rather than modelling the developmental emergence of tumors through the expression of oncogenes or deletion of functional tumor suppressors using developmentally regulated promoters, inducible systems targeting the oviductal epithelium and OSE are needed such that genetic changes can be introduced more stochastically without the requirement for surgical intervention. The initiation of tumorigenesis in the adult mouse would not only better reflect the origin of ovarian cancer in women, but it also offers a more relevant examination of the interplay of the immune system with emerging tumors [78]. In addition, controlled timing of tumorigenesis in the adult mouse would allow some flexibility in studying the disease as a function of age, including manipulation of the hormonal status of the mice either through hormonal supplementation [74] or induction of menopause [98]. This would provide an enhanced relevance of GEM of EOC, but the challenges remain on how to effectively target the appropriate cells using available technologies.

## Endnotes

^a^ All animal experiments described in this study were performed according to the Guide to the Care and Use of Experimental Animals established by the Canadian Council on Animal Care.

## Abbreviations

*Amh*: Anti-Müllerian hormone; *Amhr2*: Anti-Müllerian hormone type 2 receptor; EC: Endometrioid carcinoma; *Fshr*: Follicle stimulating hormone receptor; HGSC: High grade serous carcinoma; *LacZ*: β-galactosidase; LGSC: Low grade serous carcinoma; LMS: leiomyosarcoma; MISIIR: Müllerian inhibiting substance type 2 receptor; *Ovgp1*: oviduct specific glycoprotein; OSE: Ovarian surface epithelium; SV40TAg: SV40 early region; TAg: SV40 large T antigen.

## Competing interests

The authors declare that they have no competing interests.

## Authors’ contributions

KG, LFG and EMGP carried out the characterization of the Tg(Amhr2-SV40TAg)1Bcv transgenic line. KG participated in the design of the studies and the draft of the manuscript. BCV conceived of the study and participated in the preparation of the manuscript. All authors read and approved the final manuscript.
